# Evaluating the effects of low-dose simulated galactic cosmic rays on murine hippocampal-dependent cognitive performance

**DOI:** 10.3389/fnins.2022.908632

**Published:** 2022-12-06

**Authors:** Pilar Simmons, Madison Trujillo, Taylor McElroy, Regina Binz, Rupak Pathak, Antiño R. Allen

**Affiliations:** ^1^Division of Radiation Health, University of Arkansas for Medical Sciences, Little Rock, AR, United States; ^2^Department of Pharmaceutical Sciences, University of Arkansas for Medical Sciences, Little Rock, AR, United States; ^3^Department of Neurobiology and Developmental Sciences, University of Arkansas for Medical Sciences, Little Rock, AR, United States; ^4^Department of Aging, University of Florida, Gainesville, FL, United States

**Keywords:** galactic cosmic radiation, central nervous system, space radiation, Mars, risk

## Abstract

Space exploration has advanced substantially over recent decades and plans to increase the duration of deep space missions are in preparation. One of the primary health concerns is potential damage to the central nervous system (CNS), resulting in loss of cognitive abilities and function. The majority of ground-based research on space radiation-induced health risks has been conducted using single particle simulations, which do not effectively model real-world scenarios. Thus, to improve the safety of space missions, we must expand our understanding of the effects of simulated galactic cosmic rays (GCRs) on the CNS. To assess the effects of low-dose GCR, we subjected 6-month-old male BALB/c mice to 50 cGy 5-beam simplified GCR spectrum (^1^H, ^28^Si, ^4^He, ^16^O, and ^56^Fe) whole-body irradiation at the NASA Space Radiation Laboratory. Animals were tested for cognitive performance with Y-maze and Morris water maze tests 3 months after irradiation. Irradiated animals had impaired short-term memory and lacked spatial memory retention on day 5 of the probe trial. Glial cell analysis by flow cytometry showed no significant changes in oligodendrocytes, astrocytes, microglia or neural precursor cells (NPC’s) between the sham group and GCR group. Bone marrow cytogenetic data showed a significant increase in the frequency of chromosomal aberrations after GCR exposure. Finally, tandem mass tag proteomics identified 3,639 proteins, 113 of which were differentially expressed when comparing sham versus GCR exposure (fold change > 1.5; *p* < 0.05). Our data suggest exposure to low-dose GCR induces cognitive deficits by impairing short-term memory and spatial memory retention.

## Introduction

The vision for space exploration is focused on the eventual colonization of the moon and Mars (Tinganelli et al., 2021; Zhang Z. et al., 2021), which is the goal of the NASA Artemis program. Currently there are many spacecrafts and satellites rotating around earth where they are shielded from the much higher risk of radiation damage in deep space. The Earth’s magnetosphere protects spacecrafts in Low Earth Orbit (LEO) where radiation levels are lower and can be reduced by adequate shielding materials. As the interest in deep space exploration increases, more studies are being conducted to understand how to safely establish human presence on the Moon and Mars. The Artemis mission serves as an opportunity to establish a permanent human presence beyond low Earth orbit (LEO) (Mhatre et al., 2022). Returning to the moon will provide a means to test, refine, and develop new technologies that will extend our understanding of deep space, eventually taking us to Mars (Cahill et al., 2021). The long duration of future space missions, increased distance from earth, and prolonged exposure to ionizing radiation from galactic cosmic rays (GCRs) present a new set of challenges for crew members (Cahill et al., 2021). The major particles that makeup the GCR spectra include hydrogen, helium, carbon, oxygen, neon, silicon, calcium, and iron (Norbury et al., 2016). GCR is the most dominant, unavoidable source of radiation that astronauts will experience on deep space missions. Current estimates suggest astronauts will experience 10-fold higher GCR exposure than when on the International Space Station located within the Earth’s magnetosphere (Krukowski et al., 2021). The high penetrating power of GCR will require advances in shielding strategies to attenuate exposure on these deep space missions. In preparation for sending astronauts to Mars, researching the potential health risks associated with GCR exposure will lead to a better understanding on how to protect astronauts in the deep space environment.

Among the vast number of health concerns for astronauts engaged in space travel, the impact on the central nervous system (CNS) is of special concern. Significant research in rodent models has shown that whole-body radiation exposure can compromise the proper function and plasticity of neural network (Parihar et al., 2020). These disruptions can lead to neurocognitive decrements and neurobehavioral declines that are detrimental to the performance and success of crew members during a space mission (Parihar et al., 2020). During space travel astronauts rely on intact cognitive function for proper decision-making skills, making it imperative to clearly understand the risks associated with GCR exposure on the CNS (Klein et al., 2021). Of the various regions of the brain that play a role in regular cognitive function, the hippocampus is crucial for spatial navigation and memory formation (Robinson et al., 2020). Rodent animal studies investigating HZE particle exposures on hippocampal-dependent behavior have found to induce memory and learning impairment depending on a variety of parameters such as dosages, irradiation timelines, and sexes (Machida et al., 2010). Data from two previous studies suggests exposure to ^16^O induces behavioral deficits at a time point of 9°months and 3°months after exposure in females (Kiffer et al., 2019; Swinton et al., 2021). Many single-ion GCR irradiation models have shown differential cognitive disruptions (Klein et al., 2021). However, the deep space radiation environment consists of a much more complex variety of species over a broad range of energies (Simonsen et al., 2020), and a clearer understanding of the consequences of GCR irradiation on CNS functionality is needed. GCR simulated research provides a more translatable approach to investigate rodent behavioral deficits related to exposure on the CNS (Schaeffer et al., 2022).

## Results

To assess the effects of galactic cosmic rays (GCRs) on cognitive performance and characterize the underlying mechanisms, we subjected mice to behavioral tests and assayed various cellular and molecular endpoints associated with learning and memory 3 months after whole-body irradiation with a 50 cGy 5-beam simplified GCR spectrum (^1^H, ^28^Si, ^4^He, ^16^O, and ^56^Fe) (Table 1).

**TABLE 1:** The particles used in the 5-beam galactic cosmic rays (GCR) spectrum along with their respective doses and dose rates.

Sample name and ion species	Delivered dose	Dose rate cGy/min
p1000	17.4998	**0.5879**
Si600	0.4998	**0.2673**
He250	8.9999	**0.4009**
O350	2.9998	**0.3786**
Fe600	0.5000	**0.1289**
P250	19.4997	**0.6217**

### Y maze

**FIGURE 1:**
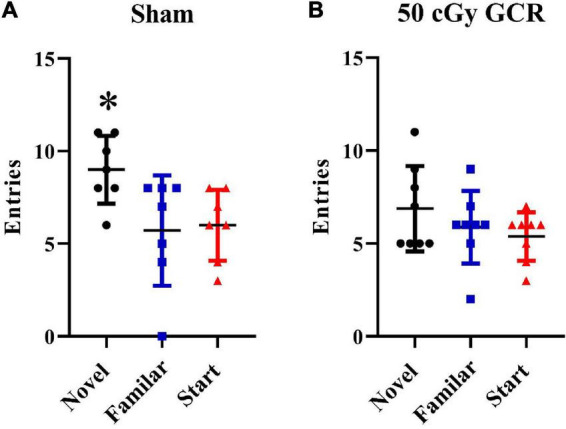
Y maze. **(A)** Sham-irradiated mice (*n* = 6) had significantly more entries into the novel arm during the testing phase of the Y maze, indicating normal spatial recognition. **(B)** Irradiated mice (*n* = 6) (50 cGy GCR) were unable to distinguish between the 3 Y-maze arms, indicating that GCR exposure impaired short-term memory. *P < 0.05.

The Y-maze paradigm takes advantage of the fact that rodents are naturally curious; thus, they will naturally orient themselves toward a novel stimulus. Preference for the novel arm indicates normal spatial recognition. Sham-irradiated animals had significantly more entries to the novel arm than the start and familiar arms, indicating normal spatial recognition [F_(2,__18)_ = 4.37, *p* < 0.05; Figure 1A]. Irradiated animals were unable to differentiate between the novel, start, or familiar arms [F_(2,__21)_ = 1.29, *P* = 0.2947; Figure 1B], indicating that GCR exposure impaired short-term memory.

### Morris water maze

#### Latency to platform

Following Y-maze testing, we used the Morris water maze to assess learning and spatial memory retention in sham-irradiated and irradiated mice. A decrease in path swim time (latency) to the platform indicates an improvement in spatial learning and memory. Mice were trained to locate a platform when it was hidden beneath the surface of the opaque water (hidden-platform training, days 1–5). Swim velocity can influence latency to the target during training sessions; however, repeated-measures ANOVA revealed there was no significant treatment-by-day interaction for velocity [F_(4,__56)_ = 0.22, *P* = 0.92]. Hidden-platform training (acquisition) requires mice to learn the location of the hidden platform based on extra-maze spatial cues. There was no significant treatment-by-day interaction [F_(4,__56)_ = 1.11, *P* = 0.36]. However, there was a significant difference in time [F_(1.97,__27.59)_ = 6.23, *P* < 0.001], meaning that these animals performed better as testing progressed.

#### Probe trials

**FIGURE 2:**
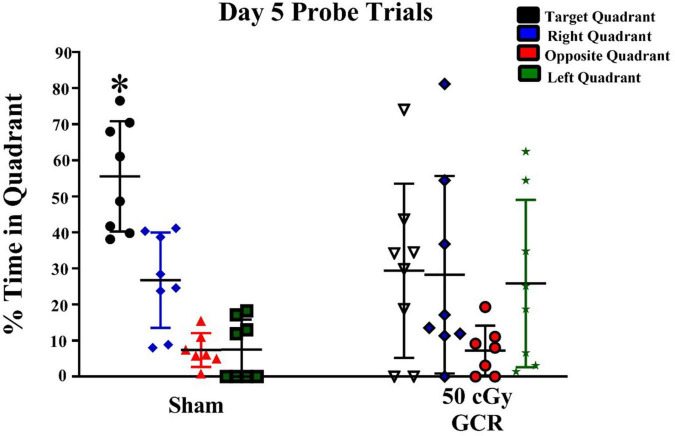
Spatial memory retention during probe trials on day 5 of Morris water maze testing. During the day of probe trials, the sham-irradiated group (*n* = 6) showed significant preference for the target quadrant; whereas, the irradiated group (*n* = 6) (50 cGy GCR) was unable to differentiate the target quadrant. *P < 0.0001.

We then conducted probe trials (platform removed) to measure spatial memory retention. BALB/c mice tend to be poor learners, so we performed the probe trials after 5 days of hidden-platform training. The sham group exhibited significant preference for the target quadrant on day 5 [F_(3,28)_ = 27.73, *P* < 0.0001; Figure 2]. In contrast, irradiated mice did not show a preference for any quadrant, indicating a lack of memory retention on day 5 [F_(3,28)_ = 0.4212, *P* = 0.739; Figure 2].

### Flow cytometry

**FIGURE 3:**
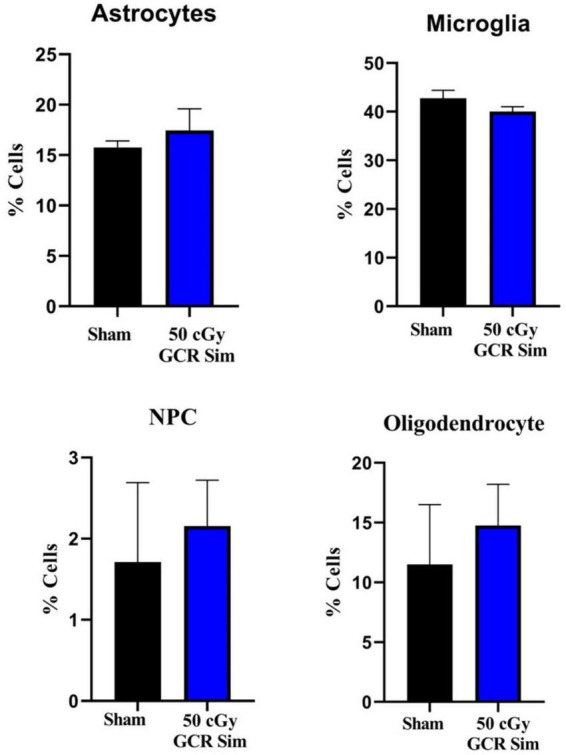
Flow cytometry was used to measure changes in astrocytes (ACSA-2–positive), neural precursor cells (PSA-NCAM–positive), microglia (CD11b-positive), and oligodendrocytes (O4-positive) of hippocampal tissue in sham-irradiated (*n* = 6) and irradiated (*n* = 6) (50 cGy GCR) mice. There were no significant changes in the proportions of each cell type between either treatment group. NPC, neural precursor cells.

Following behavioral testing, we characterized how various cellular and molecular endpoints associated with learning and memory was affected by GCR irradiation. We used flow cytometry to measure changes in astrocytes (ACSA-2–positive), neural precursor cells (PSA-NCAM–positive), microglia (CD11b-positive), and oligodendrocytes (O4-positive) of hippocampal tissue. We found No significant changes in cell counts in irradiated animals compared to sham (ACSA-2, *T* = 0.76, *P* = 0.52; PSA-NCAM, *T* = 0.39, *P* = 0.73; CD11b, *T* = 1.43, *P* = 0.29; or O4, *T* = 0.53, *P* = 0.65) (Figure 3).

### Cytogenetic analysis

**FIGURE 4:**
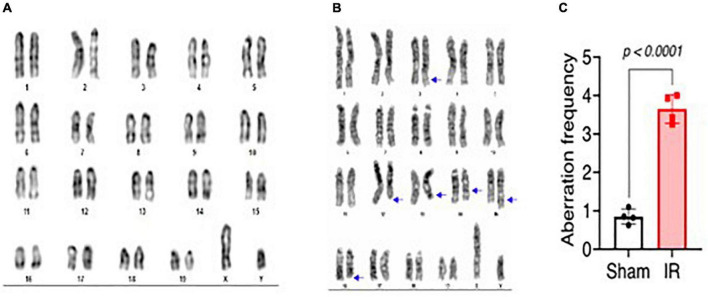
Representative photomicrographs of G-banded chromosomes from **(A)** sham-irradiated and **(B)** irradiated mice (arrows indicate change in banding pattern). **(C)** Aberration frequency in sham-irradiated (*n* = 4) and irradiated (*n* = 4) mice as estimated by observing change in banding pattern of chromosomes following G-banding. Statistical uncertainty between the groups was determined by *t*-test.

**FIGURE 5:**
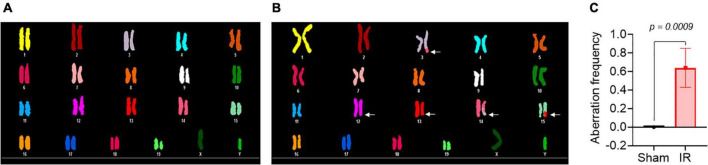
Representative photomicrographs of chromosomes following spectral karyotyping (SKY) hybridization in **(A)** sham-irradiated and **(B)** irradiated mice (arrows indicate breaks and translocations). **(C)** Aberration frequency in sham-irradiated (*n* = 4) and irradiated (*n* = 4) mice as estimated by observing change in length or color junction. Statistical uncertainty between the groups was determined by *t*-test.

**FIGURE 6:**
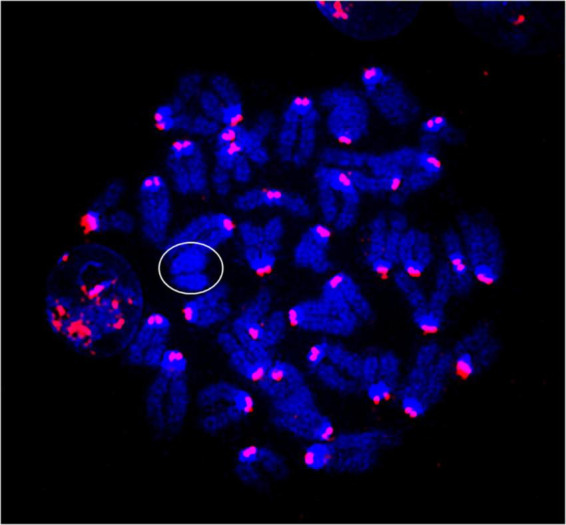
Representative photomicrographs of chromosomes following centromere hybridization (Kreatech Biotechnology B.V, Amsterdam, Netherland). Each centromere shows 2 signals except Y-chromosome (circled) in both sham-irradiated and irradiated groups. The image was captured under 63x magnification.

Metaphase chromosome spreads prepared from the bone marrow of irradiated mice (*n* = 4) or sham-irradiated mice (*n* = 4) were evaluated with G-banding, spectral karyotyping (SKY), and hybridization with fluorescent DNA probes for all centromeres. G-banding was used to determine changes in chromosome banding patterns as a result of deletions, duplications, or other rearrangements. Aberrations occurred approximately four times more frequently in irradiated mice than in sham-irradiated mice (irradiated: 193 aberrations/54 metaphase spreads vs. sham: 42/49) (Figure 4). We then used SKY analysis to validate the G-band results. Taking into consideration the complexity of analysis and cost effectiveness, samples from animals in each experimental group were combined for SKY hybridization. In the irradiated group, SKY analysis revealed a total of 9 aberrations in 14 metaphase spreads, while no aberrations were observed in 9 metaphase spreads from the sham group (Figure 5). There were no aberrations involving centromeres in either group. Notably, the centromere signal was missing on the Y-chromosome, resulting in 39 centromere signals for each cell (Figure 6), representative image of centromere hybridization).

### Proteomics

**FIGURE 7:**
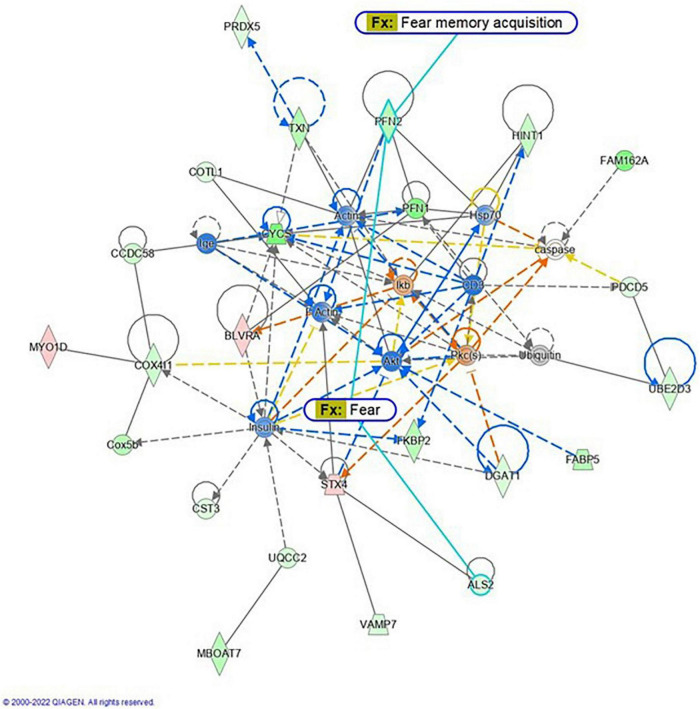
Graphic representation of mouse hippocampus protein network 1, identified by Ingenuity Pathway Analysis (IPA) as being affected by sham irradiation compared to galactic cosmic rays (GCR) irradiation. Functions associated with the network include free-radical scavenging, cellular assembly and organization, and cellular function and maintenance. The network is overlayed with the disease and function tool to display the 2 key molecules involved with behavior. The node color indicates expression value, and color intensity indicates degree of up-or down-regulation: red indicates upregulation, and green indicates downregulation. Gray nodes are dataset molecules that were not significantly expressed and therefore did not pass the IPA analysis cutoff. Uncolored nodes were not part of our dataset but were incorporated into the pathway based on evidence stored in the Ingenuity Knowledge Base. Known direct and indirect interactions between network proteins, as well as the direction of the interaction, are indicated by arrows or blocked lines. Reproduced with a license obtained from QIAGEN.

**FIGURE 8:**
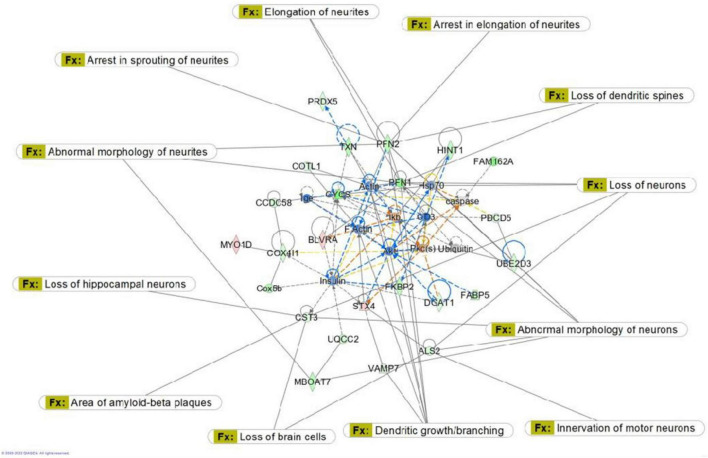
Graphic representation of mouse hippocampus protein network 1, identified by Ingenuity Pathway Analysis (IPA) as being affected by sham irradiation compared to galactic cosmic rays (GCR) irradiation. Functions associated with the network include free-radical scavenging, cellular assembly and organization, and cellular function and maintenance. The network is overlayed with the disease and function tool to display the 14 key molecules involved with cell morphology. Reproduced with a license obtained from QIAGEN.

**FIGURE 9:**
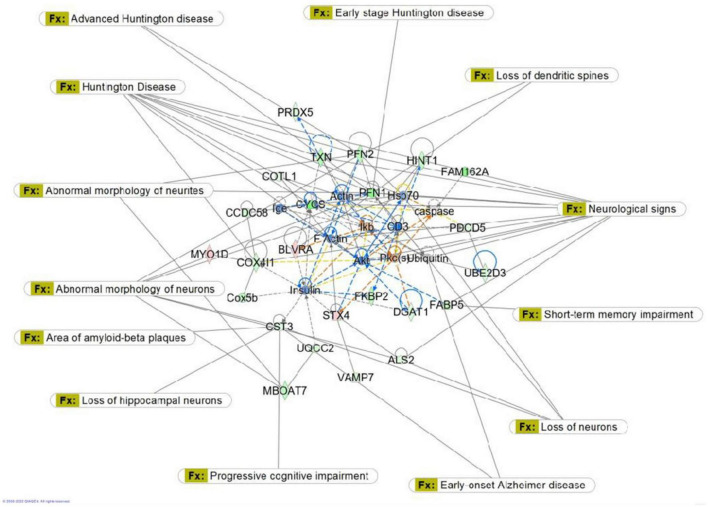
Graphic representation of mouse hippocampus protein network 1, identified by Ingenuity Pathway Analysis (IPA) as being affected by sham irradiation compared to galactic cosmic rays (GCR) irradiation. Functions associated with the network include free radical-scavenging, cellular assembly and organization, and cellular function and maintenance. The network is overlayed with the disease and function tool to display the 15 key molecules involved with neurological disease.

**FIGURE 10:**
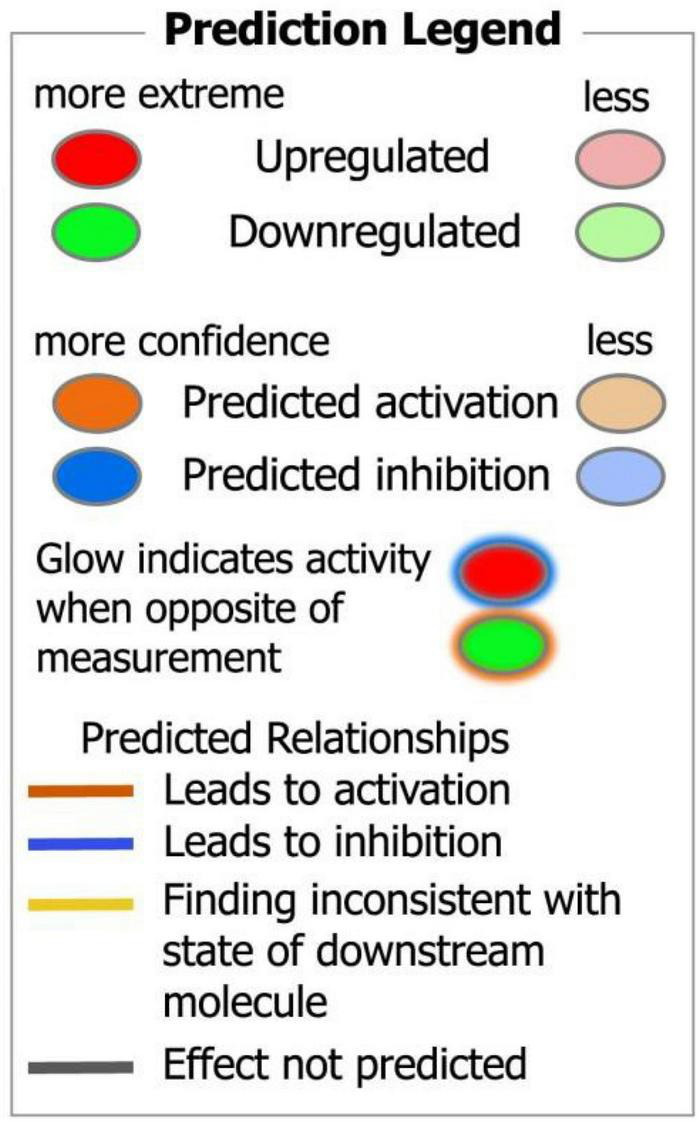
Ingenuity pathway analysis (IPA) legend of protein networks and predicted interactions.

**FIGURE 11:**
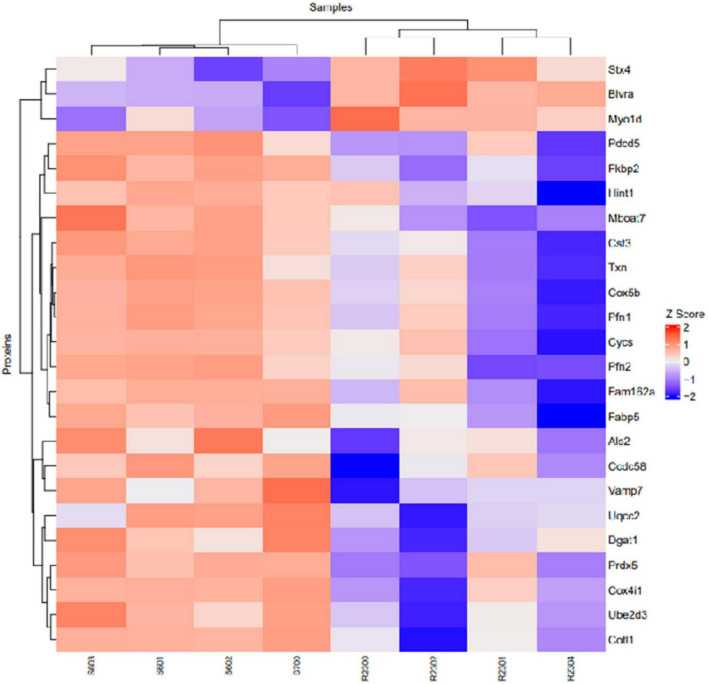
Heatmap of proteins identified in network 1 with their corresponding samples. S, Sham; R, Radiation.

**FIGURE 12:**
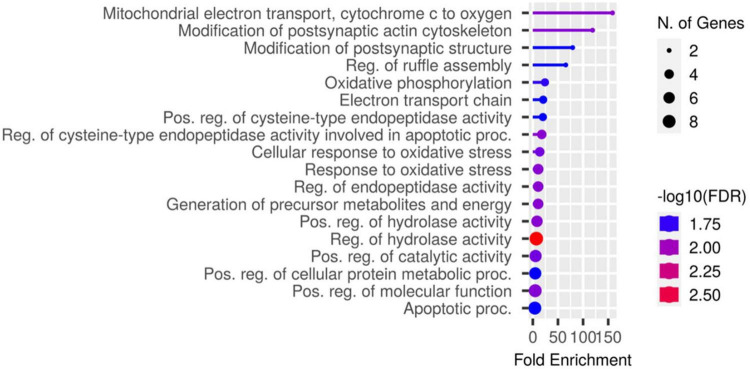
A chart identifying the GO biological processes for the genes identified in network 1.

Proteomic analysis was performed using hippocampal tissue to obtain an understanding of the proteins expressed in potential pathways and networks associated with sham irradiated groups versus GCR irradiated groups. We conducted Ingenuity Pathway Analysis (IPA) with the datasets of differentially expressed proteins and focused on the top network. IPA is a web-based software application that provides insightful analysis of proteomics data by providing networks of differentially expressed proteins. The top network from our data had functions associated with free-radical scavenging, cellular assembly and organization, and cellular function and maintenance. We utilized the disease and function overlay tool on the top protein network to provide insight on potential proteins associated with behavior, cell morphology and neurological disease between the two treatment groups. When overlayed with behavior, proteins from network 1 were associated with fear memory acquisition (Figure 7). The cell morphology overlay was associated with functions related to loss of dendritic spines, elongation of neurites and loss of hippocampal neurons (Figure 8). The neurological disease overlay display functions related to short term memory impairment, Huntington disease and early set Alzheimer’s disease (Figure 9). Tandem mass tag proteomics identified 3,639 proteins, 113 of which were differentially expressed when comparing sham versus GCR exposure (fold change > 1.5; *p* < 0.05). IPA uses a network of the differentially expressed proteins with distinct colors that represent predictions of upregulation and downregulation (Figure 10). IPA was able to provide information on the interaction between the list of proteins with associated functions within the top five networks (Tables 2, 3). From this list 24 of the focus proteins for network 1 were displayed in a heatmap (Figure 11). Most of the sham samples had a positive z score while much of the irradiated group had a negative z score. We also performed a GO analysis on the biological processes for the proteins of network 1 (Figure 12). Two of the biological processes with a higher fold was associated with postsynaptic structures.

**TABLE 2:** Top 5 ingenuity pathway analysis (IPA) networks proteins associated with sham versus galactic cosmic rays (GCR) treatments.

Network 1	Associated network functions: Cellular assembly and organization, cellular function and maintenance, free radical scavenging Number of “focus molecules” in the network: 24 IPA score: 51 Network proteins: Actin, Akt, ALS2, BLVRA, caspase, CCDC58, CD3, COTL1, COX4I1, Cox5b, CST3, CYCS, DGAT1, F Actin, FABP5, FAM162A, FKBP2, HINT1, Hsp70, Ige, Ikb, Insulin, MBOAT7, MYO1D, PDCD5, PFN1, PFN2, Pkc(s), PRDX5, STX4, TXN, UBE2D3, Ubiquitin, UQCC2, VAMP7
Network 2	Associated network functions: Connective tissue disorders, hematological disease, organismal injury, and abnormalities Number of “focus molecules” in the network: 23 IPA score: 48 Network proteins: ADCY, ATP5MF, ATP5MG, ATP6V1G1, Calcineurin protein(s), CAMK1, CIAPIN1, CIRBP, Creb, CTSV, ERBB4, ERK1/2, FKBP1A, HBA1/HBA2, Hbb-b1, Hbb-b2, hemoglobin, ISCA1, Mitochondrial complex 1, MLST8, NADH dehydrogenase, NDUFA6, NDUFB3, NDUFB4, NDUFB6, p70 S6k, PDGF BB, Pka, PPP3R1, PVALB, RALB, Secretase gamma, SH3BGRL, Sos, TGM2
Network 3	Associated network functions: Cancer, protein synthesis, RNA damage and repair Number of “focus molecules” in the network: 22 IPA score: 46 Network proteins: 60S ribosomal subunit, ANKFY1, ASNS, Calmodulin, Ck2, CSTF2, DYNLL1, DYNLT3, estrogen receptor, G3BP1, GABARAPL2, H1-0,H2AZ2, Histone h4, LARP1, Lh, NDUFAF4, NFkB (complex), PCP4, PI3K (complex), PLC, PRKAR1B, PRPF19, PYM1, RAS, RNA polymerase II, Rnr, RPL23, RPL30, RPL35A, RPS12, RPS15A, SNU13, SUB1, Tgf beta
Network 4	Associated network functions: Lipid metabolism, molecular transport, small molecule Biochemistry Number of “focus molecules” in the network: 14 IPA score: 25 Network proteins: 2210010C04Rik, ACOT13, ARL15, Arxes1/Arxes2, CNIH2, CROT, CYC1, FABP, FABP7, FANCD2, FUNDC2, GLP1R, HTT, Mup1 (includes others), MYDGF, Ndufs5, PCNA, PCTP, PGS1, PLXNB3, PPARA, RFX4, SEC11C, Slc27a, SLC27A4, SNCA, SPCS3, SPOCK2, STMN3, TMBIM4, TUBAL3, U2af1, UQCRB, VDAC3, WDR45
Network 5	Associated network functions: Cancer, cell-to-cell signaling and interaction, organismal injury and abnormalities Number of “focus molecules” in the network: 13 IPA score: 23 Network proteins: ATP5MD, C12orf57, C6orf136, CDH1, CDH17, CRIM1, CSNK2B, ESR2, GLIPR2, GOLGA7, ITGB1, LMTK2, LYPLAL1, MAN1A1, MRPS10, MRTFB, N6AMT1, Nebl, NUDT16L1, PEAK1, PIGK, PPP1CC, PXN, RAPH1, RBBP9, SCYL3, SEC11A, SEMA3B, SSBP1, TMEFF1, TMEM120A, TMEM87A, TRAPPC6B, TWF2, ZDHHC5

IPA generates these networks of proteins contained using a proprietary algorithm to generate connectivity and interaction. IPA calculates a *p*-score [-log10(*p*-value)] calculated by Fisher’s exact test. The *p*-score indicates the probability of finding the focus molecules from the experimental dataset in a network from IPA’s Global Molecular Network.

**TABLE 3:** List of proteins from Tables 1–5 with their symbol and gene name.

2210010C04Rik	Acyl-CoA thioesterase 13
60S ribosomal subunit*	
ACOT13	ADP ribosylation factor like GTPase 15
Actin*	
ADCY*	
Akt*	
ALS2	Alsin Rho guanine nucleotide exchange factor
ANKFY1	Ankyrin repeat and FYVE domain containing 1
ARL15	Adipocyte-related X-chromosome expressed sequence 2
Arxes1/Arxes2	Cornichon family AMPA receptor auxiliary protein 2
ASNS	Asparagine synthetase (glutamine-hydrolyzing)
ATP5MD	ATP synthase membrane subunit k
ATP5MF	ATP synthase membrane subunit f
ATP5MG	ATP synthase membrane subunit g
ATP6V1G1	ATPase H+ transporting V1 subunit G1
BLVRA	Biliverdin reductase A
C12orf57	Chromosome 12 open reading frame 57
C6orf136	Chromosome 6 open reading frame 136
Calcineurin protein(s)*	
Calmodulin*	
CAMK1	Calcium/calmodulin dependent protein kinase I
Caspase*	
CCDC58	Mitochondrial matrix import factor 23
CD3*	
CDH1	Cadherin 1
CDH17	Cadherin 17
CIAPIN1	Cytokine induced apoptosis inhibitor 1
CIRBP	Cold inducible RNA binding protein
Ck2*	
CNIH2	Carnitine O-octanoyltransferase
COTL1	Coactosin like F-actin binding protein 1
COX4I1	Cytochrome c oxidase subunit 4I1
Cox5b	Cytochrome c oxidase subunit 5B
Creb*	
CRIM1	Cysteine rich transmembrane BMP regulator 1
CROT	Cytochrome c1
CSNK2B	Casein kinase 2 beta
CST3	Cystatin C
CSTF2	Cleavage stimulation factor subunit 2
CTSV	Cathepsin V
CYC1*	
CYCS	Cytochrome c, somatic
DGAT1	Diacylglycerol O-acyltransferase 1
DYNLL1	Dynein light chain LC8-type 1
DYNLT3	Dynein light chain Tctex-type 3
ERBB4	Erb-b2 receptor tyrosine kinase 4
ERK1/2*	
ESR2	Estrogen receptor 2
estrogen receptor*	
F Actin*	
FABP	Fatty acid binding protein 7
FABP5	Fatty acid binding protein 5
FABP7	FA complementation group D2
FAM162A	Family with sequence similarity 162 member A
FANCD2	FUN14 domain containing 2
FKBP1A	FKBP prolyl isomerase 1A
FKBP2	FKBP prolyl isomerase 2
FUNDC2	Glucagon like peptide 1 receptor
G3BP1	G3BP stress granule assembly factor 1
GABARAPL2	GABA type A receptor associated protein like 2
GLIPR2	GLI pathogenesis related 2
GLP1R	Huntingtin
GOLGA7	Golgin A7
H1-0	H1.0 linker histone
H2AZ2	H2A.Z variant histone 2
HBA1/HBA2	Hemoglobin subunit alpha 2
Hbb-b1	Hemoglobin, beta adult major chain
Hbb-b2	Hemoglobin, beta adult minor chain
Hemoglobin*	
HINT1	Histidine triad nucleotide binding protein 1
Histone h4*	
Hsp70*	
HTT	Major urinary protein 1
Ige*	
Ikb*	
Insulin*	
ISCA1	Iron-sulfur cluster assembly 1
ITGB1	Integrin subunit beta 1
LARP1	La ribonucleoprotein 1, translational regulator
Lh*	
LMTK2	Lemur tyrosine kinase 2
LYPLAL1	Lysophospholipase like 1
MAN1A1	Mannosidase alpha class 1A member 1
MBOAT7	Membrane bound O-acyltransferase domain containing 7
Mitochondrial complex 1*	
MLST8	MTOR associated protein, LST8 homolog
MRPS10	Mitochondrial ribosomal protein S10
MRTFB	Myocardin related transcription factor B
Mup1 (includes others)	Myeloid derived growth factor
MYDGF	NADH:ubiquinone oxidoreductase core subunit S5
MYO1D	Myosin ID
N6AMT1	N-6 adenine-specific DNA methyltransferase 1
NADH dehydrogenase*	
NDUFA6	NADH:ubiquinone oxidoreductase subunit A6
NDUFAF4	NADH:ubiquinone oxidoreductase complex assembly factor 4
NDUFB3	NADH:ubiquinone oxidoreductase subunit B3
NDUFB4	NADH:ubiquinone oxidoreductase subunit B4
NDUFB6	NADH:ubiquinone oxidoreductase subunit B6
Ndufs5	Proliferating cell nuclear antigen
Nebl	Nebulette
NFkB (complex)*	
NUDT16L1	Nudix hydrolase 16 like 1
p70 S6k*	
PCNA	Phosphatidylcholine transfer protein
PCP4	Purkinje cell protein 4
PCTP	Phosphatidylglycerophosphate synthase 1
PDCD5	Programmed cell death 5
PDGF BB*	
PEAK1	Pseudopodium enriched atypical kinase 1
PFN1	Profilin 1
PFN2	Profilin 2
PGS1	Plexin B3
PI3K (complex)*	
PIGK	Phosphatidylinositol glycan anchor biosynthesis class K
Pka*	
Pkc(s)*	
PLC*	
PLXNB3	Peroxisome proliferator activated receptor alpha
PPARA	Regulatory factor X4
PPP1CC	Protein phosphatase 1 catalytic subunit gamma
PPP3R1	Protein phosphatase 3 regulatory subunit B, alpha
PRDX5	Peroxiredoxin 5
PRKAR1B	Protein kinase cAMP-dependent type I regulatory subunit beta
PRPF19	Pre-mRNA processing factor 19
PVALB	Parvalbumin
PXN	Paxillin
PYM1	PYM homolog 1, exon junction complex associated factor
RALB	RAS like proto-oncogene B
RAPH1	Ras association (RalGDS/AF-6) and pleckstrin homology domains 1
RAS*	
RBBP9	RB binding protein 9, serine hydrolase
RFX4	SEC11 homolog C, signal peptidase complex subunit
RNA polymerase II*	
Rnr*	
RPL23	Ribosomal protein L23
RPL30	Ribosomal protein L30
RPL35A	Ribosomal protein L35a
RPS12	Ribosomal protein S12
RPS15A	Ribosomal protein S15a
SCYL3	SCY1 like pseudokinase 3
SEC11A	SEC11 homolog A, signal peptidase complex subunit
SEC11C*	
Secretase gamma*	
SEMA3B	Semaphorin 3B
SH3BGRL	SH3 domain binding glutamate rich protein like
Slc27a	Solute carrier family 27 member 4
SLC27A4	Synuclein alpha
SNCA	Signal peptidase complex subunit 3
SNU13	Small nuclear ribonucleoprotein 13
Sos*	
SPCS3	SPARC (osteonectin), cwcv and kazal like domains proteoglycan 2
SPOCK2	Stathmin 3
SSBP1	Single stranded DNA binding protein 1
STMN3	Transmembrane BAX inhibitor motif containing 4
STX4	Syntaxin 4
SUB1	SUB1 regulator of transcription
Symbol	Gene Name
Tgf beta	RIKEN cDNA 2210010C04 gene
TGM2	Transglutaminase 2
TMBIM4	Tubulin alpha like 3
TMEFF1	Transmembrane protein with EGF like and two follistatin like domains 1
TMEM120A	Transmembrane protein 120A
TMEM87A	Transmembrane protein 87A
TRAPPC6B	Trafficking protein particle complex subunit 6B
TUBAL3	U2 small nuclear ribonucleoprotein auxiliary factor (U2AF) 1
TWF2	Twinfilin actin binding protein 2
TXN	Thioredoxin
U2af1	Ubiquinol-cytochrome c reductase binding protein
UBE2D3	Ubiquitin conjugating enzyme E2 D3
Ubiquitin*	
UQCC2	Ubiquinol-cytochrome c reductase complex assembly factor 2
UQCRB	Voltage dependent anion channel 3
VAMP7	Vesicle associated membrane protein 7
VDAC3	WD repeat domain 45
WDR45*	
ZDHHC5	Zinc finger DHHC-type palmitoyltransferase 5

*Missing gene names were not identified in the IPA software.

## Discussion

In preparation for NASA’s decision to return to the moon and aspirations to explore Mars, there has been an increase in studies investigating the health risks associated with exposure to galactic cosmic rays (GCRs). A critical challenge that comes with deep space exploration is the venturing of astronauts outside the Earth’s protective magnetosphere; this introduces them to hazardous levels of radiation from GCRs. Historically, most behavioral and cognitive research on hippocampal function has focused on the effects of space radiation-induced health risks using monoenergetic single particle simulation (Raber et al., 2020; Simonsen et al., 2020). However, understanding the risks linked to space travel beyond low earth orbit requires a simulator that more closely mimics the deep space radiation environment. Recent developments at the NASA Space Radiation Laboratory allow investigators to use a mixture of a broad spectrum of ions delivered in one sequence (Huang et al., 2020). With such GCR simulation available for research, a growing number of studies have indicated that exposure can result in injury to the central nervous system. In this study, we investigated the effects of low-dose simulated GCR on hippocampal-dependent cognitive performance in mice; we also analyzed and compared glial cell populations, chromosomal aberrations, and differential protein expression.

A variety of rodent studies administered behavioral assays to assess cognitive impairment and identified the possibility of risks to the central nervous system from low-dose GCR exposure (Cucinotta and Cacao, 2020). In our case, we used the Y-maze to assess potential deficits in short-term memory and the Morris water maze to assess learning and spatial-memory retention. Using the novel object recognition memory assay, Klein and colleagues found that 30 cGy GCR irradiation diminished the ability of male mice to differentiate a novel object relative to control mice (Klein et al., 2021). This suggests that normal recognition memory can be disrupted by exposure to even low doses of mixed-ion GCRs (Klein et al., 2021). Using the Morris water maze, they also observed spatial reference memory deficits during the probe trial for mice exposed to GCR irradiation (Klein et al., 2021). Our study similarly suggests that a low dose (50 cGy) of GCR irradiation impairs spatial memory functions. This observation is consistent with previous single-ion studies that established that space-radiation doses significantly impair performance in a wide range of cognitive tasks (Britten et al., 2021). Another study investigated the potential effects of 75 cGy whole-body 33-ion GCR exposure on the behavioral performance of mature male mice but reported no changes in anxiety-like behavior, object recognition, or sociability (Kiffer et al., 2022). Deviations such as this provide great insight into opportunities to explore various factors such as ion species, sex, post-irradiation timeframe, and age.

Our findings indicate no significant changes in the number of hippocampal astrocytes, neural precursor cells, microglia, or oligodendrocytes. Microglia account for 10–15% of all brain cells and are key mediators of neuroinflammatory processes (Cekanaviciute et al., 2018). Although microglia do not account for a large percentage of brain cells they play a major role in inflammation of the brain which can lead to neurodegeneration. Cognitive impairment resulting from combined GCR simulation was reported to correspond with enhanced microglia activation up to 12 months after exposure (Cekanaviciute et al., 2018). There is evidence that GCR simulation induces maladaptive activation of microglia, suggesting that targeting this cell population could be an effective countermeasure for the consequences of GCR exposure (Bustos et al., 2017). A study using sequential 3-beam radiation observed increased CD68 (marker of activated microglia) levels associated with cognitive injury in female mice but not males (Raber et al., 2019). However, in a 6-ion study, there was no effect on CD68 levels in the hippocampus of either females or males irradiated with 50 cGy (Raber et al., 2020). Overall, the activation of microglia by GCR exposure may differ as a result of radiation dosage, brain region examined, and sexual dimorphism (Bustos et al., 2017).

Galactic cosmic rays (GCR) is composed of damaging high-charge and high-energy particles that compromise the function of DNA double-strand break repair machinery, leading to the formation of structural aberrations (Li et al., 2018). In one study, SKY analysis of irradiated human embryonic kidney (HEK293) cells showed dose-dependent increases in structural chromosomal aberrations, but there was no correlation between radiation dose and the formation of specific structural aberrations (Saito et al., 2013). Data from several studies suggest that radiation-induced numerical and structural chromosomal aberrations are highly complex and random in nature (Binz et al., 2019). Another study observed chromosomal aberrations by spectral karyotyping in splenic leukocytes from mice irradiated with 1 Gy and 3 Gy [31]. Likewise, we observed a significantly high aberration frequency in irradiated mice.

Here, we used a tandem mass tag proteomics approach to investigate changes in the proteome between sham-and GCR-irradiated mice. Current proteomics tools allow a better understanding of the proteome that can be beneficial in finding effective countermeasures to spaceflight-induced alteration (Rea et al., 2016). By applying Ingenuity Pathway Analysis (IPA) to our proteomics data, we were able to identify the top protein networks and canonical pathways that may be related to sham versus GCR-induced cognitive impairment. We focused in on Network 1, whose associated functions include free-radical scavenging, cellular assembly and organization, and cellular function and maintenance. The analysis provided a representative diagram of protein–protein interactions and a list of proteins that were upregulated or downregulated within the network. For example, the more intensely green-colored proteins in Figure 7 are CYCS, PFN1, and FAM162A, each representing an upregulated protein with a varying function. The CYCS gene encodes cytochrome c, which functions as a central component of the electron transport chain in mitochondria (Che et al., 2021). PFN1 (profilin 1) is best known to promote and direct actin polymerization (Murk et al., 2021). Recent findings link PFN1 to neurological diseases such as amyotrophic lateral sclerosis and Huntington’s disease (Murk et al., 2021). PFN1 is also closely associated with PFN2, as shown by the line indicating protein interaction in Figure 7. Using the “disease and function overlay” option in the IPA software, we identified key molecules associated with behavior within the network. Figure 7 indicates that PFN2, which was downregulated in the network, is linked to fear memory acquisition. Figure 8 displays an overlay that highlights the key proteins associated with cell morphology. Both PFN1 and PFN2 are associated with loss of dendritic spines, dendritic growth/branching, abnormal morphology of neurons, and abnormal morphology of neurites. PNF1 and PNF2 are both highly expressed in the hippocampus, where the brain shows high activity-dependent synaptic plasticity (Michaelsen et al., 2010). A study investigating the 2 profilin isomers showed that knocking down PFN2a significantly decreased dendrite complexity and spine numbers of hippocampal neurons, while the overexpression of PFN1 prevented the loss of spines (Michaelsen et al., 2010). Finally, FAM162A is proposed to be involved in the regulation of apoptosis and hypoxia-induced cell death of neuronal cells (Lee et al., 2004). Findings from a recent study also identified it as one of 6 novel biomarkers for Alzheimer’s progression (Zhang T. et al., 2021).

In Figure 9, we used the “disease and function overlay” feature to examine proteins in the network associated with neurological disease. CST3 was downregulated in the network. CST3 (cystatin c) is a cysteine protease inhibitor that plays an important role in brain homeostasis, and dysregulation of its inhibitory effects could alter neuroinflammatory conditions leading to neurodegeneration (Sheikh et al., 2021). In the neurological disease overlay, CST3 was associated with area of amyloid-beta plaques, early onset Alzheimer’s disease, progressive cognitive impairment, and loss of hippocampal neurons (Figure 9). Findings from one study suggested that CST3 is associated with impaired cognition in elders and that it could be a useful biomarker for cognitive function in future studies (Yaffe et al., 2008). Overall, data from our proteomics analysis is useful when researching possible countermeasures by identifying potential biomarkers within the pathway cascade and understanding protein interactions within the networks associated with sham versus GCR exposure.

## Conclusion

We used 6-month-old male BALB/c mice to assess the effects of low-dose simulated 50 cGy GCRs (^1^H, ^28^Si, ^4^He, ^16^O, and ^56^Fe) on hippocampal-dependent cognitive performance. Additionally, we measured changes in glial cell count using flow cytometry, performed a cytogenetic analysis for any chromosomal aberration detection and a proteomic analysis of the Sham group compared to GCR. The use of flow cytometry to identify the proportion of cells has been gaining popularity as a method of choice for a variety of studies (Fraker et al., 1995). Much of the popularity is due to its rapid and highly quantitative power and high throughput capabilities (Pugsley, 2017). For the Y-maze behavioral analysis we observed an impairment of short-term memory in mice exposed to GCR as compared to the sham-irradiated animals. We also observed a lack of memory retention for the irradiated mice in the MWM during the probe trials. The sham-irradiated mice were observed to have intact spatial memory retention. We found no significant changes in cell counts from flow cytometry measurements in irradiated animals compared to sham. The G-band results from the cytogenetic analysis showed chromosomal aberrations occurred four times more frequently in irradiated mice than in sham-irradiated mice. Despite the increase in mission relevant doses and GCR simulations used to investigate radiation beyond LEO on cognitive function, there is still a need to incorporate more gender comparison studies. A caveat to our work is that we only examined male mice, but future studies should include both sexes. NASA has plans to send the first woman to the Moon and more space agencies are seeking to deploy more women on their missions (Semple et al., 2020). Future research should focus on producing data from GCR exposure using both male and female rodent models to more accurately understand sex-specific differences of the effects on cognition in Mars.

## Materials and methods

### Animals and simulated galactic cosmic rays exposure

Eight-week-old male BALB/c mice were purchased from The Jackson Laboratory (Bar Harbor, ME, USA) and housed five animals per cage at the University of Arkansas for Medical Sciences (UAMS). The mice received standard rodent chow and water *ad libitum*. Once the animals were 6 months old, they were transferred to Brookhaven National Laboratory, where they were acclimated for 1 week before they were exposed to a simplified whole-body GCR simulation beam designed by NASA. The beam consisted of protons at 1,000 MeV and 250 MeV, ^28^Si at 600 MeV/n, ^4^He at 250 MeV/n, ^16^O at 350 MeV/n, and ^56^Fe at 600 MeV/n. The total dose of 50 cGy was chosen as a probable equivalent dose that an astronaut would receive during a 1.5- to 2-year deep space mission (Nelson, 2016). Sham-irradiated mice underwent the same protocol but did not receive any radiation. Two days after simulated GCR exposure, both irradiated and sham-irradiated mice were shipped back to UAMS. During the following 8-week quarantine protocol, mice received 150 ppm fenbendazole in chow. At the end of the quarantine period, mice were transferred to a non-barrier animal facility and maintained an additional 4 weeks until they were euthanized by cervical dislocation followed by decapitation for blood and tissue collection.

### Behavioral scheme

#### Y-maze

Mice were handled 5 days prior to the onset of behavioral testing. Prior to each day of testing, mice were acclimated to the testing room in their home cage for 1 h. All testing took place during the animal’s dark cycle. Mice first completed a Y-maze task to assess short-term spatial memory and exploratory activity. They were exposed in 2 trials to an apparatus composed of 3 arms: start, familiar, and novel (Gottlieb et al., 2006). Each clear, acrylic arm (45 × 15 × 30 cm) of the Y-maze contained a unique visual cue affixed to the end of the arm. The Y-maze is based on the instinctive curiosity of rodents to explore novel stimuli without positive or negative reinforcement (Dellu et al., 1992). In the first trial (i.e., training trial), mice were introduced in the start arm (facing the end of the arm) and allowed to explore the start and familiar arms for 5 min; the novel arm was blocked during the initial training trial. After a 4-h intertrial interval, the mice were reintroduced to the Y-maze for the second trial (i.e., testing trial) and allowed to explore all 3 arms for 5 min. As rodents naturally orient their head toward novel stimuli, this orienting response should habituate following subsequent exposure given intact learning and memory domains (Honey et al., 1998). Thus, cognitively intact mice should preferentially explore the novel arm. Allocation of arms was counterbalanced across trials. The arena was cleaned with 20% ethanol between every trial.

#### Morris water maze

Hippocampal-dependent spatial learning and memory were assessed with the Morris Water Maze. The maze is a 5-day task during which the mice are required to locate a hidden platform (days 1–5) on the basis of extra-maze cues (Benice et al., 2006). A circular pool (diameter: 140 cm) was filled with temperature-controlled (24°C) opaque water. Large, clearly visible cues were located on the test room curtains. For hidden-platform days, the platform did not change quadrants. Each day, there were 2 sessions with a 2-h intertrial interval, and each session consisted of 3 trials with a 10-min intertrial interval. During acquisition, each mouse was gently placed in the water maze facing the wall from one of seven locations, excluding the location immediately next to the platform (i.e., northeast and southwest starting locations were excluded for the first and second test times, respectively). If the mouse failed to find the platform within the maximum allotted time of 60 s, it was gently placed onto the platform for 20 s.

To measure spatial memory retention, probe trials were conducted following the third session on day 5. For the probe trial, the hidden platform was removed from the pool. Mice were placed in the quadrant opposite the target quadrant (previous location of the hidden platform) and allowed to swim for 60 s. The time spent in the target quadrant was compared to the time spent in the three non-target quadrants. Average swim velocity and distance to platform were also used as measures of performance (Allen et al., 2014). A CCD video camera was located above the maze for automatic behavioral analysis using EthoVision^®^ XT video tracking system (Noldus Information Technology, Leesburg, VA, USA). After the probe trial on day 5, mice were sacrificed.

### Perfusion

Mice were sedated with isoflurane and checked for a toe-pinch reflex for pain before any procedures were done. Following complete anesthesia, an incision was made below the diaphragm, and the rib cage was cut rostrally on the lateral edges to expose the heart. A small hole was cut in the left ventricle and a needle was inserted into the aorta and clamped, then the right atrium was cut to allow flow. The mice were transcardially perfused with PBS for 4–5 min or until the liver was cleared of blood. The heart and liver were monitored during perfusion to ensure the right ventricular chamber remained somewhat darker in color than the left ventricular chamber and the liver blanched when blood was replaced with PBS.

### Dissociation and flow cytometry analysis

Hippocampal tissues were dissected and dissociated by enzymatic digestion using the Neural Tissue Dissociation Kit P (Miltenyi Biotec) in combination with the gentleMACS Octo Dissociator (Miltenyi Biotec) (Trujillo et al., 2021). Single-cell suspensions obtained from neural tissue were stained with fluorochrome-conjugated antibodies for flow cytometry analysis [Anti-ACSA-2-(PE-Vio770), Anti-PSA-NCAM-(PE), Anti-CD11b-(VioBlue), and Anti-O4-(APC); Miltenyi Biotec]. FcR Blocking Reagent (mouse, Miltenyi Biotec) was always added to prevent non-specific antibody binding to Fc receptors.

### Chromosome preparation

Each mouse received 100 μL 0.5% colchicine by intraperitoneal injection 20 min before tissue harvest. Post anesthesia, the animal was sacrificed for bone marrow procurement. Samples were kept on ice until synchronization at hypotonic treatment. Briefly, each femur bone was resected, removing both ends. Bone marrow was collected into 1 ml cold buffer solution (0.5% BSA in PBS) by centrifugation at 10,000 *g* for 10 s. Bone fragments were discarded. The bone marrow cells at the bottom of the microcentrifuge tube were carefully dissociated and strained to obtain a single cell suspension. Fresh buffer was added to bring the total volume to 5 ml. Cells were centrifuged at 400 *g* for 5 min. The supernatant was removed, leaving approximately 1 ml of solution. Samples were removed from ice and thoroughly re-suspended. Cells were gradually warmed by addition of warm hypotonic solution (75°mM KCl; Gibco) in 1 ml increments to a final volume of 8 ml. Harvest tubes were placed in a 37°C water bath and maintained in the hypotonic solution for 30 min. To ensure proper transition into the fixative stage, 0.5 ml fixative (3:1 methanol:acetic acid) was added dropwise to each harvest tube. Each tube was properly capped and inverted gently to evenly incorporate the fixative into solution. Following centrifugation at 1,000 rpm for 10 min, the supernatant was removed to 1.5 ml. Each pellet was gently re-suspended, and 2 ml of 5:2 fixative was added in a dropwise manner with simultaneous agitation. An additional 6 ml of fixative was added to each harvest tube, mixed thoroughly, and the samples were allowed to rest for 20 min at room temperature. Harvest tubes were centrifuged for 8 min at 1,000 rpm, the supernatant was aspirated to 0.5 ml, and cells were re-suspended with 6 ml fresh fixative. This cycle was repeated 3 times. Cell suspensions were diluted appropriately and applied dropwise to clean microscope slides.

### G-banding

Trypsin-Giemsa staining was used to prepare G-banded chromosomes. Slides were baked overnight at 66°C and treated with 0.025% trypsin for 1°min, gently rinsed with Tyrode’s buffer (Sigma, St. Louis, MO, USA), and stained with Giemsa (Sigma) for 5°min. For karyotyping, images were acquired with a Zeiss Imazer.Z2 microscope equipped with GenASIs Case Data Manager system, version 7.2.2.40970. At least 80 well-spread randomly selected metaphase spreads were photographed and analyzed for each treatment group.

### Spectral karyotyping

We used SKY to supplement G-banding. The SKY kit from Applied Spectral Imaging (ASI, Carlsbad, CA, USA) was used according to the manufacturer’s protocol; the probe mixture and hybridization reagents were prepared as recommended. Briefly, prior to hybridization, slides were soaked in 2X SSC (Sigma) at room temperature for 5°min then dehydrated in 70, 80, and 100% ethanol for 2°min each. Slides were air dried, placed in pre-warmed denaturing solution [70% formamide (Millipore, Temecula, CA, USA) in 2X SSC] at 71°C for 1°min, then immediately dehydrated in a cold ethanol series (70, 85, and 100%) for 2°min each. Meanwhile, SKY probe was pre-warmed to 37°C for 5 min and transferred to a water bath at 80°C–81°C to denature for 7°min. Once samples were denatured and dehydrated, 10°μl denatured SKY probe was applied to the target area of the slide, covered with a 22°x°22-mm coverslip, and sealed with rubber cement. Slides were protected from light in a humidified chamber and allowed to hybridize for 48 h at 37°C. Hybridized slides were subjected to 2 washes (5 min each) with (pre-warmed to 45°C) formamide solution (50% formamide in 2X SSC) and 1X SSC. Once removed from the 1X SSC solution, the slide was allowed to drain, and 100°μl blocking reagent was applied to the target area and protected with a plastic coverslip. Meanwhile, Cy5 antibody solution (ASI) was reconstituted with filtered 4X SSC. Following a 30-min incubation at 37°C, the coverslip was carefully removed, Cy5 antibody solution was applied to the target area, protected with a fresh plastic coverslip, and returned to the 37°C incubator. Following 1°h of hybridization, the slide was subjected to a wash series (pre-warmed to 45°C) consisting of 3 jars containing 4X SSC and 0.1% Tween-20 in each one of them (5°min each). Meanwhile, Cy5.5 antibody solution (ASI) was reconstituted with filtered 4X SSC. Upon completion of the wash series, the slide was briefly dipped into distilled water to remove detergent residue, and Cy5.5 antibody solution was applied to the target area and protected with a fresh plastic coverslip. Following a 30-min incubation at 37°C and three washes with 4X SSC/0.1% Tween-20 slides were briefly dipped in distilled water. DAPI counterstain was immediately applied, and a clean glass coverslip was placed over the entire slide. Image acquisition for SKY was performed with an SD200 Spectracube (ASI) mounted on a Zeiss Imager.Z2 microscope. DAPI images were captured and then inverted and enhanced with SKY View software to produce G-band–like patterns on the chromosomes. At least 30 SKY images were captured under 63X magnification per experimental group. The visualization of all human chromosomes in different colors was achieved with spectral imaging. Spectral imaging combines fluorescence microscopy, CCD-imaging, and Fourier spectroscopy to visualize simultaneously the entire spectrum at all image points.

### Centromere hybridization

All mouse centromere FISH probe (Kreatech Biotechnology B.V, Amsterdam, Netherland) was used to detect centromeric repetitive sequences located at the primary constriction centromere of mouse chromosomes. After dehydration with ethanol series, slides were air dried, placed in pre-warmed denaturing solution [70% formamide (Millipore, Temecula, CA, USA) in 2X SSC] at 71°C for 1°min, then immediately dehydrated in a cold ethanol series (70, 85, and 100%) for 2°min each. FISH probe was denatured at 91°C for 4°min. Then, 10 μl of probe was applied to a sample area of approximately 22 × 22 mm and incubated overnight at 37°C in a humidified chamber. Next day, slides were washed with 4X SSC and 0.1% Tween-20 solution, DAPI counterstain was immediately applied, and a clean glass coverslip was placed over the slide. Images were captured with a Zeiss Imazer.Z2 microscope under 63x magnification.

### Protein isolation

The hippocampus was homogenized in RIPA buffer on ice. Homogenates were centrifuged and supernatants collected. Protein concentrations were determined with the Bradford assay kit (Thermo Fisher) following the manufacturer’s instructions.

### Tandem mass tag proteomics analysis

Tandem mass tag (TMT) technology is a powerful tool for precise and accurate quantitative proteomics. This method has been used widely to characterize protein expression profiles and investigate and compare functional changes at the protein level. The protocol involves extraction of proteins from cells or tissues followed by reduction, alkylation, and digestion. Samples from each experimental group were labeled with 1 of 6 isobaric tags of the TMT reagent. Resulting peptides were pooled at equal concentrations before fractionation and data acquisition. The TMT-labeled samples were analyzed by LC–MS/MS. In an MS1 scan, same-sequence peptides from the different samples appear as a single unresolved additive precursor ion. Fragmentation of the precursor ion during MS/MS (MS2) yields sequence-informative b- and y-ions, and further fragmentation by MS3 (SPS) provides quantitative information as distinct masses between m/z 126 and 131 representing the “different” reporter ions. The reporter ion intensity indicates the relative amount of peptide in the mixture that was labeled with the corresponding reagent.

### Bioinformatics analysis

Ingenuity Pathway Analysis (Qiagen) was used to investigate affected signaling pathways involving proteins of interest. The ROAST method was used to investigate unidirectional and bidirectional regulation of significant proteins.

## Data availability statement

The original contributions presented in this study are publicly available. This data can be found here: http://proteomecentral.proteomexchange.org/cgi/GetDataset?ID=PXD035933 (Project accession: PXD035933 and Project doi: 10.6019/PXD035933).

## Ethics statement

This animal study was reviewed and approved by University of Arkansas for Medical Sciences Institutional Animal Care and Use Committee.

## Author contributions

PS and AA: conceptualization, investigation, and writing—original draft preparation. PS, AA, RB, and RP: methodology. PS, MT, TM, RB, and RP: data curation. PS, RP, and AA: writing—review and editing. AA: funding acquisition. All authors have read and agreed to the published version of the manuscript.
